# Prehospital stroke scale (FAST PLUS Test) predicts patients with intracranial large vessel occlusion

**DOI:** 10.1002/brb3.1087

**Published:** 2018-08-07

**Authors:** Daniel Václavík, Michal Bar, Lukáš Klečka, David Holeš, Martin Čábal, Robert Mikulík

**Affiliations:** ^1^ AGEL Research and Training Institute Ostrava Vítkovice Hospital Ostrava Czech Republic; ^2^ Department of Neurology and Psychiatry Faculty of Medicine Ostrava University Ostrava Czech Republic; ^3^ Comprehensive Stroke Centre University Hospital Ostrava Ostrava Czech Republic; ^4^ Primary Stroke Centre City Hospital Ostrava Ostrava Czech Republic; ^5^ Emergency Health Service Ostrava Ostrava Czech Republic; ^6^ Jessenius Faculty of Medicine in Martin Comenius University in Bratislava Martin Slovak Republic; ^7^ Department of Neurology St. Anne's University Hospital Brno Czech Republic; ^8^ Faculty of Medicine Masaryk University Brno Czech Republic; ^9^ International Clinical Research Centre Stroke Research Program St. Anne's University Hospital Brno Czech Republic

**Keywords:** large vessel occlusion stroke, paramedics, triage test

## Abstract

**Background and Purpose:**

Mechanical thrombectomy (MT) is indicated for the treatment of large vessel occlusion (LVO) stroke. MT should be provided as quickly as possible; therefore, a test identifying suspected LVO in the prehospitalization stage is needed to ensure direct transport to a comprehensive stroke center (CSC). We assume that patients with clinically severe hemiparesis have a high probability of LVO stroke. We modified the FAST test into the FAST PLUS test: The first part is the FAST test and the second part evaluates the presence of severe arm or leg motor deficit. This prospective multicenter study evaluates the specificity and sensitivity of the FAST PLUS test in detecting LVO stroke.

**Methods:**

Paramedics were trained through e‐learning to conduct the FAST PLUS test.

All prehospital suspected stroke patients who were administered the FAST PLUS test were included. Demographics, National Institutes of Health Stroke Scale (NIHSS) score, brain computed tomography (CT), and CT angiography (CTA) were recorded. Sensitivity, specificity, positive predictive value (PPV), negative predictive value (NPV), and receiver operating curve (ROC) area for LVO were calculated.

**Results:**

The study included 435 patients. LVO were found in 124 patients (28%). Sensitivity was 93%, specificity was 47%, PPV was 41%, NPV was 94%, and ROC area for ICA/MCA occlusion was 0.65. Intracerebral hemorrhage (ICH) was identified in 48 patients (11%).

**Conclusion:**

We found that the FAST PLUS test had a high sensitivity for LVO stroke. Of the 435 patients, 41% were all directly transported to a CSC based on positive FAST PLUS test scores and were potential candidates for MT.

## 1 INTRODUCTION

Mechanical thrombectomy (MT) is indicated for the treatment of large vessel occlusion (LVO) stroke(Goyal et al., 2016), and the time to administer mechanical thrombectomy is a very important factor for good clinical outcome (Saver et al., 2016). MT is offered only in comprehensive stroke centers (CSC), as an endovascular team is required for the procedure. MT should be provided as quickly as possible; therefore, a test identifying a suspected occlusion in the prehospitalization stage is needed to ensure direct transport to a CSC.

There are two options for transporting patients to a CSC. In the “drip‐and‐ship” option, all suspected stroke patients are transported to primary stroke centers (PSC). Patients diagnosed with stroke and LVO are then transported to a CSC for MT. This accelerates thrombolysis administration but delays endovascular procedures.

The second option is “mothership”: Patients with suspected LVO are directly transported to a CSC. This option accelerates endovascular procedures but may delay intravenous thrombolysis and may overload the CSC with misdiagnosed patients, including stroke mimics and non‐LVO stroke patients.

In recent years, efforts have been made to develop a prehospital test that would enable the identification of patients with LVO for direct transport to a CSC. This test should be sufficiently sensitive and specific; it should be simple for paramedics and tested in prehospital practice (Michel, 2017).

A series of such tests have been published, but there has not yet been any implementation in prehospital practice (Hastrup, Damgaard, Johnsen, & Andersen, 2016; Katz, McMullan, Sucharew, Adeoye, & Broderick, 2015; Lima et al., 2016; Nazliel et al., 2008; Scheitz et al., 2017; Singer et al., 2005). Only the RACE and Cincinnati tests for LVO have been performed at a prehospital level (McMullan et al., 2017; Pérez de la Ossa et al., 2014).

Both severe hemiparesis and monoparesis have been demonstrated as the most identifiable symptoms of LVO stroke (Nakajima et al., 2004). We assume that the patients with clinically severe hemiparesis have a high probability of LVO stroke. Therefore, the FAST test was modified to the FAST PLUS test: the first part is the FAST test and the second part evaluates only the presence of severe arm or leg motor deficit (Kleindorfer et al., 2007). The aim of this prospective observational cohort study was to determine the specificity, sensitivity, positive predictive value (PPV), and negative predictive value (NPV) of the FAST PLUS test as administered by paramedics for LVO and confirmed by CT angiography (CTA).

## 2 METHODS

### 2.1 Ethical approval of the study protocol

The study protocol was approved by the Ethics Committee of University Hospital Ostrava (Ostrava, Czech Republic), Approval Number 82/2016. All patients provided written informed consent to participate in the study.

ClinicalTrials.gov Identifier: NCT03072524.

### 2.2 FAST PLUS test

The FAST PLUS test has two parts. The first part is the FAST test, which is employed in all possible cases of stroke occurrence. This test consists of the following items: Facial palsy (0–1), any failure of Arm motor function (0–1), and Speech (scored 0–1). The FAST test is considered positive if the score is at least one.

**Figure 1:**
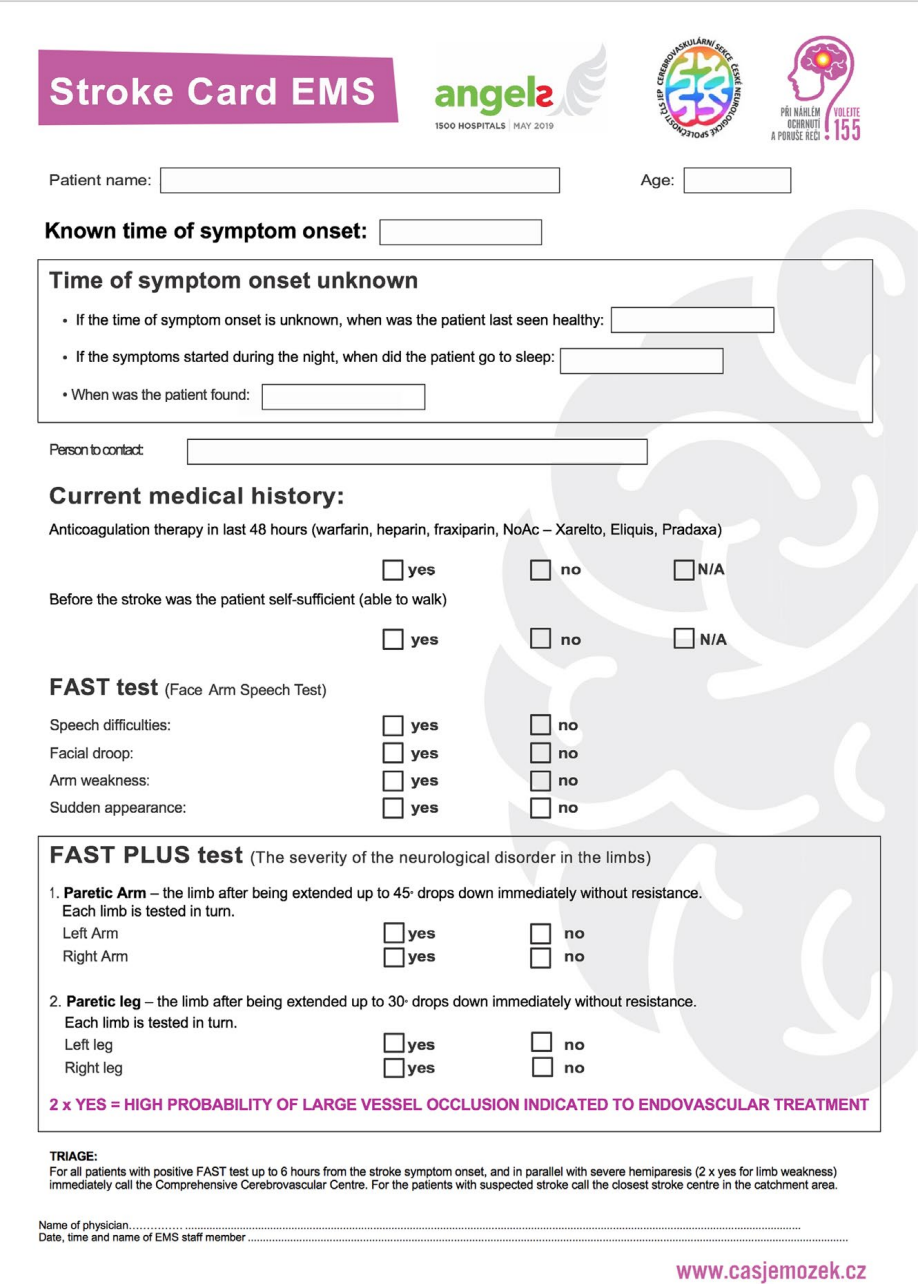
Stroke card and FAST PLUS test

The second part of the FAST PLUS test evaluates only the presence of severe arm or leg motor deficit (scored 0–1). An NIHSS score of 3 or 4 for arm or leg is considered a severe deficit. The FAST PLUS test results are considered positive when there is a positive general FAST test score and severe paresis of a leg or an arm or both. A completely new version of the Stroke Card was created using the FAST PLUS test criteria (Figure 1).

### 2.3 Training of paramedics

In previous practice, paramedics selected suspected stroke patients according to their FAST test results. For this study, paramedics were trained via e‐learning to conduct the FAST PLUS test. For their further education, three video recordings were used in order to demonstrate the examination for motor deficit in the lower and upper limbs. The first video shows a patient with complete hemiparesis; the NIHSS score was 4 for both limbs. The second video shows a patient with severe hemiparesis, with an NIHSS score of 3 for both limbs. The third video shows a patient with mild hemiparesis, with an NIHSS score of 2 for both limbs. A certified neurologist performed the NIHSS scoring.

### 2.4 Study population

Prehospital patients with suspected stroke (FAST test positive) were transported by emergency medical services to one of the three stroke centers in Ostrava (Czech Republic) according to their territory. Mechanical thrombectomy (MT) is provided at two of these centers. The catchment area of the centers was 637,584 inhabitants. Patients were transported according to the stroke triage protocol established in the Czech Republic.

Inclusion criteria for this study were as follows: (a) Suspected acute stroke patient admitted to one of the three stroke centers; (b) FAST PLUS test evaluation by paramedics; and (c) CT and CTA evaluations.

The exclusion criterion was suspected stroke with more than 12 hr from symptom onset.

The following baseline parameters were recorded: gender, age, FAST PLUS test results, total NIHSS score during admission, NIHSS score for arms, NIHSS score for legs, brain CT results, an occlusion of the middle cerebral artery (MCA) part M1/2 or of the intracranial internal carotid artery (ICA), etiology other than ischemic stroke, onset of stroke within 6 hr, number of patients with systemic thrombolysis, and mechanical recanalization.

A neurologist verified the accuracy of the FAST PLUS test data entered by the paramedics. In addition, a written paper record for each patient was submitted to a neurologist along with the FAST PLUS test results.

Sensitivity, specificity, PPV, and NPV were calculated.

### 2.5 Statistical analysis

Basic descriptive statistics were used for the final evaluation and statistical analysis. The FAST PLUS test was evaluated in terms of sensitivity and specificity calculations with a 95% confidence interval. The area under the receiver operating curve (ROC) was also calculated. Statistical tests were evaluated with a 5% significance level.

The Stata version 14 software was used for the statistical analysis. ROC and areas under the ROC (c‐statistics) were calculated as measures of the FAST PLUS test's predictive ability for LVO. An ideal prediction produces a c‐statistic of 1.00; precision no better than chance is associated with a c‐statistic of ≤0.50.

## 3 RESULTS

Over the 10‐month study period, 1,605 patients with suspected stroke were transported to stroke centers by emergency medical services. This reflects 252 strokes per 100,000 inhabitants in a catchment area of 637,584 inhabitants. Of the 1605 patients, 899 patients (56%) arrived within 12 hours of symptom onset; 435 of these patients (47%) had been administered a FAST PLUS test. These 435 patients were included in the study.

**Figure 2:**
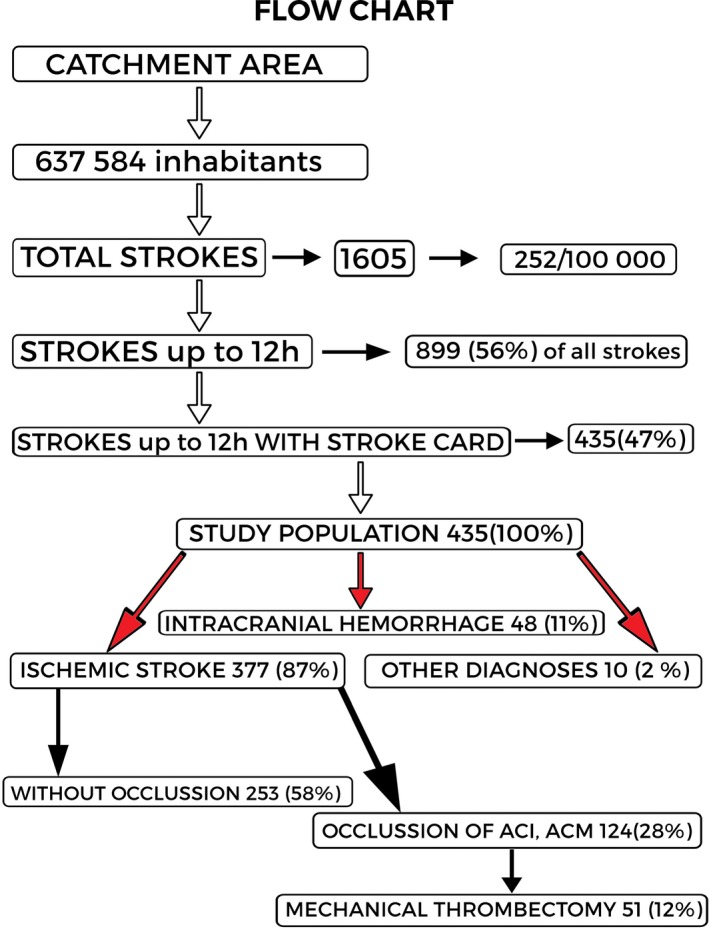
Participant flow chart

Figure 2 presents a flow chart of the patients.

Men formed 51% of the study population; the average age was 73 years (median 74). Baseline data are shown in Table 1. Of the 435 patients who were administered a FAST PLUS test, 377 (87%) had ischemic stroke, 48 patients (11%) had intracranial hemorrhage, and 10 patients (2%) had a final nonstroke diagnosis (brain tumors, epileptic seizures, central nervous system inflammation, and migraine). The mean NIHSS score of all 435 patients was 8.6; for ischemic stroke patients, it was 8.3, and for hemorrhagic stroke 13.

**Table 1:** Baseline patient characteristics

Data file characteristics		No (%)	Average	Median	*SD*	Range
No. of patients		435 (100)				
Age			73	74	12	24‐97
Women		220 (49)				
Men		215 (51)				
Ischemic stroke patients (ISP)		377 (87)				
Other diagnosis		58 (13)				
Hemorrhage		48 (11)				
NIHSS			8.6	6	7.2	0‐33
NIHSS arm			1.9	1	1.6	0‐4
NIHSS leg			1.5	1	1.5	0‐4
NIHSS	Ischemia		8.3	6		
NIHSS arm	Ischemia		1.8	1		
NIHSS leg	Ischemia		1.5	1		
NIHSS	Another dg.		13	15		
NIHSS arm	Another dg.		2.8	3.5		
NIHSS leg	Another dg.		2.9	3		
CT proven ischemia in ISP		99 (23)				
Stroke symptoms up to 6 hr		317 (72)				
ICA, MCA occlusion in ISP		124 (28)				
Mechanical recanalization in ISP		51 (12)				
Systematic thrombolysis in ISP		156 (36)				

CT: computed tomography; ICA: internal carotid artery; ISP: ischemic stroke patients; MCA: middle cerebral artery; NIHSS: National Institutes of Health Stroke Scale.

LVO was identified in 124 patients (28%). In 99 patients (23%), early ischemic changes were visible on CT; 156 patients (36%) received systemic thrombolysis; and 51 (12%) received mechanical recanalization.

The FAST PLUS test results are shown in Table 2. Positive FAST PLUS test results were returned for 280 patients (64%): 233 (54%) ischemic patients and 47 (11%) nonischemic stroke patients.

**Table 2:** FAST+ test results

	No (%)
FAST PLUS positive test	280 (64)
FAST PLUS positive test; upper and lower limbs	234 (54)
FAST PLUS positive test; upper or lower limb	46 (10.6)
Lower limb disability	2 (0.5)
Upper limb disability	44 (10.1)
FAST PLUS positive test—only ischemic stroke patients	233 (54)
FAST PLUS positive test‐other diagnosis	47 (11)
No. of improved patients during the transport	38 (9)

Of the patients with positive FAST PLUS test results, 234 (54%) patients had severe deficits of both extremities; 46 patients (10.6%) had only one severe extremity deficit: an upper extremity in 44 cases (10.1%) and a lower extremity in 2 cases (0.5%).

Table 3 shows the results of sensitivity, specificity, PPV, NPV, and ROC area.

**Table 3:** FAST+ test results—sensitivity, specificity, PPV, NPV

	LVO present	LVO absent
FP positive	115	165
FP negative	9	146
Sensitivity	% (CI 95)	93 (87‐97)
Specificity	% (CI 95)	47 (39‐50)
PPV	% (CI 95)	41 (35‐47)
NPV	% (CI 95)	94 (88‐97)
ROC area		0.65

LVO: large vessel occlusion; NPV: negative predictive value; PPV: positive predictive value; ROC: receiver operating curve.

Most of the LVO patients (115/124; 93%) had positive FAST PLUS test results, which shows a high sensitivity of 93% (95% CI 87–97) and NPV (94%). Specificity was 47% (95% CI 39–50), PPV was 41% (95% CI 35–47), and ROC area for ICA/MCA occlusion was 0.65.

## 4 DISCUSSION

Our study found that the specificity and PPV of the FAST PLUS test were 47% and 41%. This corresponds to the results of the published tests G‐FAST (39%) and CPSSS (40%) (Table 4) (Katz et al., 2015; Scheitz et al., 2017).

**Table 4:** Comparison of prehospital scale tests

Test	Sensitivity	Specificity	Evaluating items in 2 steps (yes/no)	Evaluation of gaze	Tested by paramedics
FAST PLUS all	92	44	Yes	No	Yes
FAST PLUS ischemic	93	49	Yes	No	Yes
RACE	85	68	No	Yes	Yes
LAMS	81	89	No	No	Not for LVO
FAST ED	60	89	No	Yes	No
PASS	66	83	Yes	Yes	No
S3ISS	67	92	No	Yes	No
CPSSS	83	40	Yes	Yes	No
C‐STAT	71	70	Yes	Yes	Yes, 58 patients
G‐FAST	89	39	Yes	Yes	No

In fact, our study found that a simple test such as the presence of hemiparesis can identify 41% (PPV = 41%) of patients with LVO. Practically, when the test is applied to a population with a 28% prevalence of LVO, four of 10 FAST PLUS test positive patients are directly transported to a comprehensive stroke center could be expected to have LVO. The acceptability of this number depends on several considerations, such as the capacity of prehospital services and CSCs, because 60% of patients would not benefit from such transport.

The real value of LVO prevalence is not yet precisely known; it ranges from 4.7% to 24% (Dozois et al., 2017; Rai et al., 2017). As PPV strongly depends on LVO prevalence, we provided expected PPV for populations with 10% and 20% LVO prevalence (Table 5). Such a prevalence could be expected if the FAST PLUS test is applied more generally to a less select population. In such populations, PPV would decrease to 30% and 16%, which would limit the usefulness of FAST PLUS test. On the other hand, of 165 false‐positive cases, 47 patients had intracerebral hemorrhage; such patients may still benefit from direct transport to a CSC.

**Table 5:** Correlation between prevalence of LVO stroke and PPV of FAST PLUS test

Prevalence of LVO (%)	28	20	10
PPV (%) (95% CI)	41 (35.0‐47.0)	30.4 (28.0‐31.9)	16.3 (14.8‐17.9)

The presented test has a higher sensitivity and NPV than other tests (Table 4) (Hastrup et al., 2016; Katz et al., 2015; Lima et al., 2016; McMullan et al., 2017; Nazliel et al., 2008; Pérez de la Ossa et al., 2014; Scheitz et al., 2017; Singer et al., 2005). We found that the FAST PLUS test had a high sensitivity of 93% and high NPV of 94%. Therefore, if the direct transport to CSC is selected, the majority of patients with LVO occlusion could be identified.

In practice, the FAST PLUS test, with its high sensitivity and NPV, is suitable for “mothership” transport systems in areas with a short distance between the PSC and a CSC that has a sufficient capacity for systemic thrombolysis in patients without LVO.

There are 32 PSC (1 per 300,000 inhabitants) and 12 CSC (1 per 900,000) in the Czech Republic. The FAST PLUS test seems suitable for countries with similar networks of stroke care, without delaying systemic thrombolysis or overloading the CSC (Tomek et al., 2017).

The FAST PLUS test focusing only on the evaluation of limb paresis is very simple. Severe hemiparesis and monoparesis have been demonstrated as the most identifiable symptoms of LVO stroke (Fischer et al., 2005; Kalita et al., 2013). The FAST PLUS test evaluates each item with only a two‐degree scale (yes/no), in contrast to most tests using scales with three or more degrees (Table 4).

The FAST PLUS test was administered in real stroke care practice in the Czech Republic at the prehospital level. We enrolled 435 patients with median NIHSS scores of 8 into our study. The number of patients and NIHSS scores is comparable to the RACE study (Pérez de la Ossa et al., 2014).

The majority of published tests are based on retrospective analyses of the NIHSS scores of patients with LVO. However, these tests have not yet been validated at the prehospital level (Hastrup et al., 2016; Katz et al., 2015; Lima et al., 2016; Nazliel et al., 2008; Scheitz et al., 2017; Singer et al., 2005). The best prehospital data are currently provided by the RACE test: 357 patients, which is the most extensive of all available tests. The LAMS test has been used in prehospital care for stroke identification, but not for predicting LVO (Nazliel et al., 2008). In a pilot study of the Cincinnati test, untrained paramedics tested only 58 patients (Table 4) (McMullan et al., 2017; Pérez de la Ossa et al., 2014).

Our study has several limitations.

Only 47% of the stroke patients were given a FAST PLUS test. This is comparable to the RACE study (McMullan et al., 2017) Other studies were performed retrospectively or with small numbers of patients (Hastrup et al., 2016; Katz et al., 2015; Lima et al., 2016; McMullan et al., 2017; Nazliel et al., 2008; Pérez de la Ossa et al., 2014; Singer et al., 2005).

We cannot rule out selection bias or the higher prevalence of LVO than could be expected when the test is applied in the future. Patients were enrolled only after admission to hospital, which explains the low percentage of stroke mimics (2%).

Another limitation of our study was that paramedics were trained only once via e‐learning. Three video recordings with different levels of hemiparesis were presented, without further testing. The training was not obligatory, so not all paramedics were trained. A more thorough education process could lead to better results.

The clinical impact of “mothership” or “drip‐and‐ship” transport systems has not yet been assessed. According to published studies, secondary transport significantly prolongs the time from stroke onset to recanalization (Mørkenborg, Steglich‐Arnholm, Holtmannspötter, & Krieger, 2015; Zhao et al., 2017).

The influence of the FAST PLUS test or other triage stroke tests on the time from stroke onset to MT or to systemic thrombolysis in non‐LVO patients was not studied and needs to be evaluated in further studies.

## 5 CONCLUSION

We found that the FAST PLUS test is highly sensitive to the presence of LVO; the majority of patients with LVO could be identified. However, PPV is moderate, so less than half of the patients identified by FAST PLUS test could eventually have LVO. Nevertheless, the test is very simple, and its results could be improved by better training of paramedics.

## CONFLICT OF INTEREST

The authors report no conflicts of interest. The authors themselves are responsible for the content and composition of the manuscript.
